# Impaired Tertiary Dentin Secretion after Shallow Injury in *Tgfbr2*-Deficient Dental Pulp Cells Is Rescued by Extended CGRP Signaling

**DOI:** 10.3390/ijms25136847

**Published:** 2024-06-21

**Authors:** Monica Stanwick, Fatma Fenesha, Ahmed Hamid, Khushroop Kang, Dane Kanniard, Irene Kim, Nicholas Mandarano, Fernanda L. Schumacher, Sarah B. Peters

**Affiliations:** 1 Division of Biosciences, College of Dentistry, The Ohio State University, Columbus, OH 43210, USA; stanwick.10@osu.edu (M.S.); fenesha.1@osu.edu (F.F.); hamid.55@osu.edu (A.H.); kang.1162@osu.edu (K.K.); kanniard.8@osu.edu (D.K.); kim.8244@osu.edu (I.K.); 2 Division of Biostatistics, College of Public Health, The Ohio State University, Columbus, OH 43210, USA; mandarano.4@osu.edu (N.M.); schumacher.313@osu.edu (F.L.S.)

**Keywords:** transforming growth factor beta, calcitonin gene-related peptide, tertiary dentin, neuropeptide, dental pulp cells, pulp biology, fibrosis

## Abstract

The transforming growth factor β (TGFβ) superfamily is a master regulator of development, adult homeostasis, and wound repair. Dysregulated TGFβ signaling can lead to cancer, fibrosis, and musculoskeletal malformations. We previously demonstrated that TGFβ receptor 2 (*Tgfbr2*) signaling regulates odontoblast differentiation, dentin mineralization, root elongation, and sensory innervation during tooth development. Sensory innervation also modulates the homeostasis and repair response in adult teeth. We hypothesized that *Tgfbr2* regulates the neuro-pulpal responses to dentin injury. To test this, we performed a shallow dentin injury with a timed deletion of *Tgfbr2* in the dental pulp mesenchyme of mice and analyzed the levels of tertiary dentin and calcitonin gene-related peptide (CGRP) axon sprouting. Microcomputed tomography imaging and histology indicated lower dentin volume in *Tgfbr2^cko^* M1s compared to WT M1s 21 days post-injury, but the volume was comparable by day 56. Immunofluorescent imaging of peptidergic afferents demonstrated that the duration of axon sprouting was longer in injured *Tgfbr2^cko^* compared to WT M1s. Thus, CGRP+ sensory afferents may provide *Tgfbr2*-deficient odontoblasts with compensatory signals for healing. Harnessing these neuro-pulpal signals has the potential to guide the development of treatments for enhanced dental healing and to help patients with TGFβ-related diseases.

## 1. Introduction

The TGFβ superfamily of ligands and receptors regulates multiple cellular processes throughout the body, including proliferation, migration, differentiation, development, inflammation, matrix secretion, and calcification [1,2,3,4,5,6]. Dysregulated TGFβ signaling can interrupt one or many of these processes and lead to diseases such as cancer, fibrosis, and dento-skeletal malformations [7,8,9,10,11,12]. Importantly, TGFβ is considered the primary factor responsible for fibrosis [13,14,15,16], indicating that its balance is crucial to maintain organ health and wound healing. Therefore, TGFβ signaling is a therapeutic target for these diseases. For example, pirfenidone entered the market in 2014 to treat idiopathic pulmonary fibrosis [17], and several additional agents targeting TGFβ are currently in clinical trials [16,18,19,20,21,22,23]. Some, such as Vactosertib, are likely to enter the market in the near future [24]. However, due to the pleiotropic nature of TGFβ, these drugs often induce adverse side effects, which have hampered the therapeutic progress for many agents. It is important to continue investigations of TGFβ signaling in multiple contexts, including its selective silencing in adult organs, in order to address how it might be specifically targeted for wound healing and inflammation, and to address how to overcome the adverse side effects of TGFβ-inhibiting drugs. In dentistry, this knowledge could help enhance or regulate the calcification of pulp following injury, potentially prevent pulp stones, and help preserve the pulp tissue with age.

Previous research has shown that TGFβ signaling regulates the proliferation and differentiation of mineralizing cells, including osteoblasts and odontoblasts [25,26,27,28,29,30,31,32,33]. Several reports have shown that the secretion and mineralization of dentin involves the TGFβ superfamily, predominantly mediated through TGFβ receptors I and II, which are expressed in both odontoblasts and dental pulp cells [25,26,31,34,35]. Dentinogenesis continues throughout the lifetime of an animal, with primary dentin laid down during tooth development, secondary dentin slowly secreted throughout adulthood, and tertiary dentin secreted in response to injury or infection [36,37]. If *Tgfbr2* is deleted in mature odontoblasts in a developing tooth, the cells lose their polarity, resulting in ectopic matrix formation resembling fibrotic tissue, osteodentin formation, and even pulpal obliteration [31,38]. In our recent report, we found that *Tgfbr2* in dental pulp fibroblasts and odontoblasts regulates the postnatal neuro-pulpal development of mouse molars [25,26,28]. These hypoinnervated, hypomineralized teeth sometimes also demonstrated fibrotic tissue within the pulp [28]. Based on our studies, we posited an intimate link between odontoblast signaling and axon sprouting orchestrated by signals downstream of *Tgfbr2* that leads to an innervated and mineralized tooth organ.

Sensory afferents have been shown to maintain tooth homeostasis, and denervation of the pulp leads to fibrosis that is evident within 2 weeks in rats and continually worsens with time [39]. These observations indicate that peptidergic signals are crucial to pulp health. Furthermore, a shallow dentin injury without pulp exposure has been shown to initiate the expression of neurotrophic factors, such as nerve growth factors (NGF), by odontoblasts [26,31,35]. This triggers a neuroinflammatory response in which sensory afferents sprout and secrete calcitonin gene-related peptide (CGRP) [27,28] to activate odontogenic activities for dentin repair [40,41]. Research showing that CGRP+ sensory afferents sprout during dentin injury was first published more than three decades ago [42], but conflicting data have recently emerged regarding the involvement of peptidergic signaling from the sensory neurons in reactionary and/or reparative dentinogenesis. One group reported that CGRP has minimal effects on the expression of differentiation markers in dental pulp stem cells (DPSCs) in vitro unless paired with Sonic Hedgehog (Shh) [41]. However, they also reported that CGRP alone promotes DPSC mineralization [43]. Another group found that applying CGRP promoted pulpal healing in ferrets, with increased levels of tertiary dentin and osteodentin [44]. Another group reported that CGRP inhibits mineralization [45]. It should be noted that these reports experimented directly on DPSCs, while we and others have shown an intimate, bi-directional communication between the DPSCs and trigeminal neurons [40,46,47,48,49,50,51,52,53,54,55]. Importantly, a recent report indicated that CGRP neurons are crucial to musculoskeletal wound healing involving multiple cell populations [56], suggesting that in vivo studies are likely necessary to understand how the nervous system regulates healing, including in the teeth.

Taken together, these data led us to hypothesize that TGFβ signaling likely regulates dentin healing and that the absence of *Tgfbr2* would result in aberrant or reduced tertiary dentin formation. Since TGFβ signaling in the dental pulp cells promotes axon sprouting during tooth development [25,57], we hypothesized that this also occurs during dentin repair. In the present study, we utilized the tetracycline-responsive Osterix-Cre; *Tgfbr2*^f/f^ mouse model to perform a timed deletion of *Tgfbr2* in the odontoblasts and dental pulp cells during a shallow dentin injury and investigated whether this altered the CGRP+ axon sprouting and tertiary dentin secretion. Our results indicate that sensory afferents assist the compromised, *Tgfbr2*-deficient pulp cells with dentin healing and that targeting the CGRP-signaling complex could be an effective way to prevent fibrotic responses and promote healing in damaged teeth.

## 2. Results

### 2.1. Tgfbr2^cko^ Model Characterization

**Figure 1:**
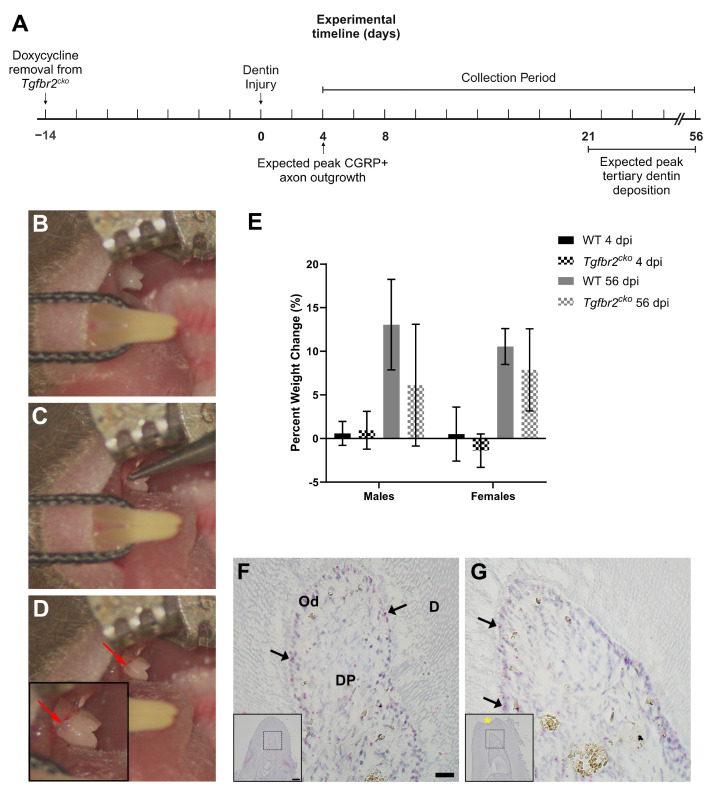
Experimental timeline for dentin injury and model validation. (**A**) Timeline of dentin injury activities, beginning with doxycycline removal for *Tgfbr2^cko^* mice 14 days prior to dentin injury and the collection of tissue on specific days post-injury (dpi). (**B**–**D**) Creation of the dentin injury. Pre-injury (**B**), injury in process with a #1/16 carbide round bur (**C**), and post-injury (**D**), with an inset showing a higher magnification image of the injury (red arrows). (**E**) Percent weight change post-dentin injury in male and female WT and *Tgfbr2^cko^* mice at 4 dpi and 56 dpi. There was no significant difference in weight pre- to post-dentin injury in either genotype, sex, or dpi. (**F**,**G**) *Sp7* (Osterix) in situ hybridization of 4 dpi *Tgfbr2^cko^* control (**F**) and injured (**G**) mice to confirm Osterix-Cre expression (N = 4). Osterix was being actively transcribed in both control and injured *Tgfbr2^cko^* sections (black arrows). Insets show lower magnification of sections, with dotted lines outlining the main images of (**F**) and (**G**). Dentin injury is marked with the yellow * in the inset of (**G**). Od = odontoblasts, DP = dental pulp, D = dentin. Scale bars = 10 μm (**F**) and 100 μm (F inset).

To confirm that dentin injury did not affect eating behaviors or body weight, which could affect tooth healing, mice were weighed pre- and post-surgery. We found no significant differences in weights between WT and *Tgfbr2^cko^* male and females (Figure 1E).

Mice were raised from in utero until 2 weeks prior to surgery with doxycycline-infused chow. To infer the activity of Cre after transitioning to standard chow, we performed ISH for *Sp7* (Osterix) on injured and control teeth. ISH revealed active transcription of *Sp7*, indicating that there was Cre expression and therefore conditional deletion *Tgfbr2* in the *Tgfbr2^cko^* mice (Figure 1F–G).

### 2.2. Tertiary Dentin Deposition Is Delayed in Tgfbr2^cko^ M1s

**Figure 2:**
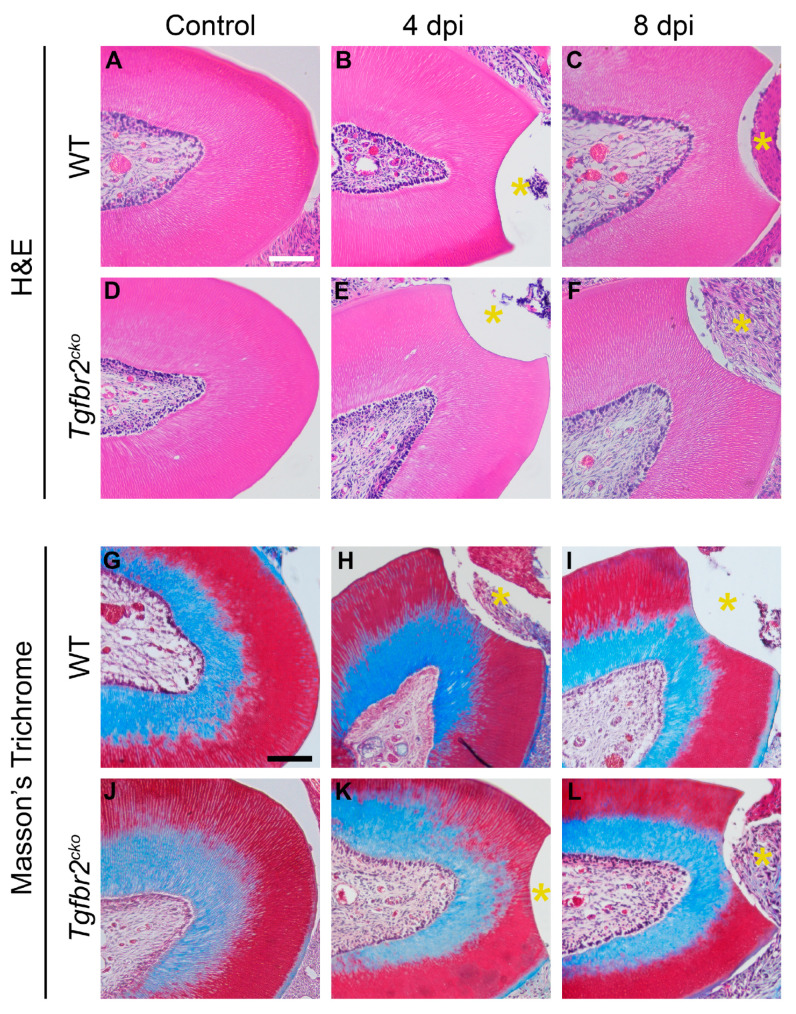
Histological analyses of 4 and 8 dpi M1s. No tertiary dentin was present at 4 or 8 dpi in WT (**A**–**C**,**G**–**I**) or *Tgfbr2^cko^* (**D**–**F**,**J**–**L**) M1s as depicted by H&E (**A**–**F**) and Masson’s trichrome staining (**G**–**L**). The yellow asterisks indicate the area drilled due to dentin injury. Scalebars in (**A**,**G**) = 50 µm. N = 6/genotype/time point.

We did not find evidence of tertiary dentin at 4 and 8 days post-injury (dpi) irrespective of mouse genotype (Figure 2).

**Figure 3:**
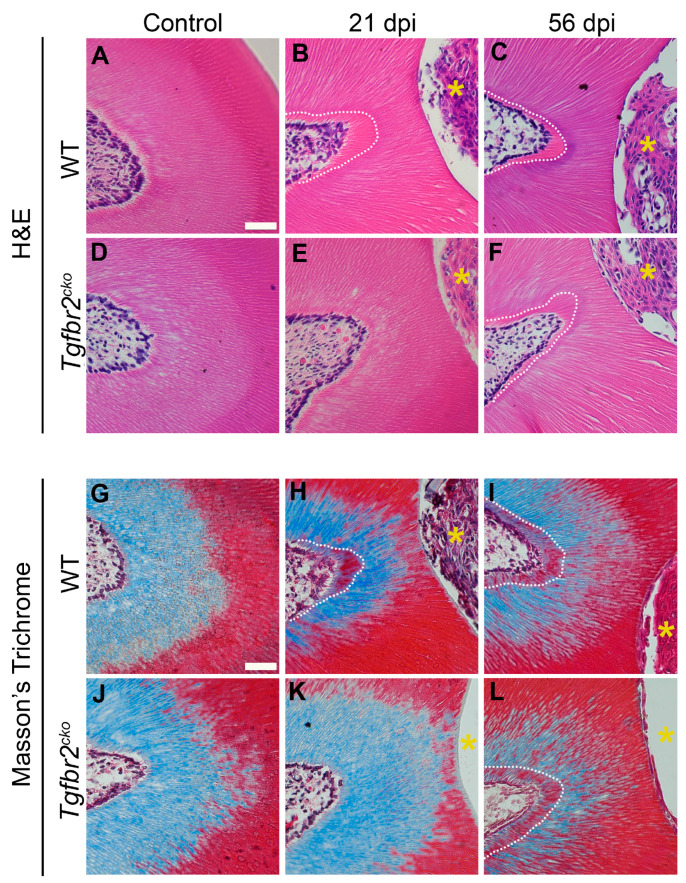
Histology of tertiary dentin formation. (**A**–**F**) H&E and (**G**–**L**) Masson’s trichrome stained coronal sections (7 µm thick) of control, 21 dpi, and 56 dpi M1s. Tertiary dentin formation was not demonstrated at 21 dpi in the *Tgfbr2^cko^* mice (**E**,**K**) compared to WT mice (**B**,**H**). Comparable tertiary dentin was formed by 56 dpi in both the *Tgfbr2^cko^* (**F**,**L**) and WT (**C**,**I**) mice. The yellow asterisks indicate the area drilled due to dentin injury. White dotted lines demarcate the tertiary dentin border. Scale bar (shown in (**A**)) = 10 µm. N = 6-8/genotype/time point.

While we found tertiary dentin in injured WT molars at 21 dpi, we did not find any in *Tgfbr2^cko^* molars (Figure 3B,E,H,K). The WT molars did not demonstrate increases in tertiary dentin beyond the 21 dpi time point (Figure 3B,C,H,I). Intriguingly, we found tertiary dentin in the *Tgfbr2^cko^* mice at 56 dpi similar to what we saw in the WT mice (Figure 3C,F,I,L). Histological staining depicted polarized odontoblasts in both uninjured and injured molars in both genotypes, with tubular tertiary dentin. Masson’s trichrome generally stained the outer, or more external, regions of the dentin in Biebrich scarlet and the inner regions in aniline blue at the cemento–enamel junction. The regions of tertiary dentin were also stained in Biebrich scarlet.

**Figure 4:**
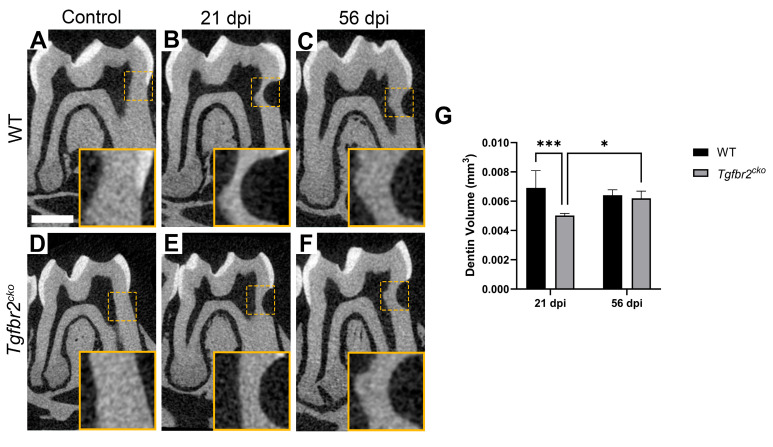
Micro-CT analysis of tertiary dentin formation. (**A**–**G**) Micro-CT analysis (6 µm resolution) of control, 21 dpi, and 56 dpi M1s from all groups. Yellow dotted lines indicate inset images in (**A**–**F**) that represent the ROIs used for quantifications. There was a significantly lower dentin volume in *Tgfbr2^cko^* mice at 21 dpi compared to WT mice (**B**,**E**,**G**), but at 56 dpi, the tertiary dentin volume was comparable between genotypes (**C**,**F**,**G**). Scale bars = 1 mm. In (**G**), * indicates *p* < 0.05 and *** indicates *p* < 0.001 by mixed-effects 2-way ANOVA.

We also performed micro-CT to quantify changes in dentin between the genotypes within the injury area. We did not find any evidence of tertiary dentin until 21 dpi. At 21 dpi, we found differences in dentin volume within the ROI between the WT and *Tgfbr2^cko^* mice (Figure 4B,E,G; *p* < 0.001). Intriguingly, we found that the tertiary dentin volume levels in the *Tgfbr2^cko^* mice at 56 dpi were equivalent to what was present in the WT at 21 and 56 dpi (Figure 4B,C,E,F,G; *p* < 0.05). We did not find any changes in dentin density (Supplementary Materials, Figure S1, Table S2). 

### 2.3. CGRP+ Axon Sprouting Is Elevated in Tgfbr2^cko^ M1s

**Figure 5:**
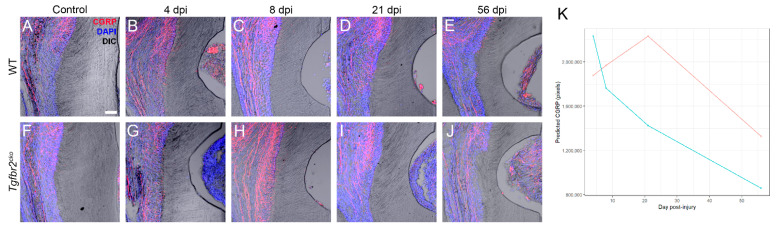
CGRP+ axon sprouting in response to dentin injury. (**A**–**J**) Representative maximum projections of confocal images (20 μm thick) showing the CGRP+ (red) axon outgrowth in control mice and WT and *Tgfbr2^cko^* M1s collected at 4–56 dpi (N = 4–8/group). DAPI is labelled in blue. Dentin is visible with Differential Interference Contrast (DIC). The injury is visible as a half-moon on the right of each frame (**B**–**E**,**G**–**J**) via DIC imaging. In some images, gingiva is apparent in the injured area. *Tgfbr2^cko^* CGRP+ axon sprouting increased significantly between 4 and 21 dpi compared to that in the WT mice (**B**,**D**,**G**,**I**). At 56 dpi, there were no significant differences in sprouting between the genotypes (**E**,**J**). Scalebar in (**A**) = 50 μm. (**K**) Prediction of CGRP pixels in response to injury for each genotype fit from a generalized estimating equation model.

We imaged and quantified the pixel density of CGRP+ axons in uninjured and 4, 8, 21, and 56 dpi M1s from WT and *Tgfbr2^cko^* mice to determine the extent of peptidergic axon sprouting in response to injury (Figure 5A–J, Figure S2). The analysis was based on imaging CGRP with confocal microscopy and converting the z-stacks into a maximum projection for 20 μm sections in the area of the injury. Similar areas were chosen for control samples. The z-stacks were auto thresholded and pixel density of CGRP was then calculated with ImageJ for control areas for 4, 8, and 21 time points for both genotypes. We then utilized generalized estimating equations (GEEs) [58] (Figure 5K, Table S1) to analyze the data while accounting for the repeated measures. Model prediction results of CGRP are presented based on the genotype, injury status, and time point, and a detailed statistical analysis is included in Supplementary Materials, Table S1. We found that the predicted CGRP at 4 dpi was not statistically different between WT and *Tgfbr2^cko^* M1s (Figure 5B,G). However, the change in CGRP from 4 to 21 dpi differed between the two genotypes for injured mice (*p* = 0.004), with the predicted model indicating a slower decline in CGRP in the injured *Tgfbr2^cko^* mice, as shown by the red line in Figure 5K. By 56 dpi, the CGRP seems to stabilize in both injured genotypes. No significant differences were observed in CGRP+ sprouting by sex across all time points and genotypes (Supplementary Materials, Table S2). Our results suggest that CGRP sprouting in response to injury remains elevated longer in *Tgfbr2^cko^* compared to WT molars and predicts a different healing trajectory.

## 3. Discussion

We previously reported that *Tgfbr2* in dental pulp fibroblasts and odontoblasts regulates the postnatal neuro-pulpal development of mouse molars [25,26,28]. In this report, we sought to determine whether the signaling downstream of *Tgfbr2* in the developing dental pulp mesenchyme also regulates reactionary dentinogenesis and odontoblast secretion of neurotrophic signals that promote axon sprouting. Since conditional deletion of *Tgfbr2* in the Osteocalcin-Cre model results in pulpal obliteration [31], and deletion in the Osterix-Cre model results in late postnatal death [25,26,27,28], we utilized the tetracycline-responsive element in the Osterix-Cre mouse model to isolate conditional deletion of *Tgfbr2* to the time period immediately before the dentin injury and continuing through the studies on the injury response. Our ISH studies of Sp7 indicated that there was *Tgfbr2* deletion in the dental pulp, confirming the validity of our model. Unexpectedly, we found that while tertiary dentin secretion was delayed in the *Tgfbr2^cko^* molars, the levels of tertiary dentin were eventually equivalent to those demonstrated in WT control mice. In addition, the axon sprouting in *Tgfbr2^cko^* mice was equivalent to that in the WT mice at 4 dpi but remained elevated for a longer period of time following injury than in the WT mice. Even though recent research has demonstrated that CGRP signaling differs between male and female models of pain, we did not find a difference in CGRP+ axon sprouting between the sexes [59], indicating that sex did not play a primary role in reactionary dentinogenesis in our models. Together, our results suggest that the sensory afferents may have secreted CGRP for a longer period in the *Tgfbr2^cko^* mice to assist with dentin repair.

It is common practice to etch dentin during endodontic procedures, but etching and some pulp capping materials can induce cellular damage and hypersensitivity [60,61,62,63,64,65,66]. In mouse models of dentin injury, etching can cause severe damage and hyperactive responses that do not replicate the scenarios one would encounter in a clinical setting [40]. In our experiment, we used a low-speed drill to prevent exposure to high heat during the procedure and did not etch the dentin afterward. This protected the nearby axon terminals and underlying odontoblast layer, as evidenced in our confocal images of the afferents and histological images of the odontoblast layers. Since axon coverage can drastically vary between ROIs, and a thin histological section could misrepresent the true nature of the wound response. We based our quantifications on confocal imaging of the CGRP+ axon sprouting of two consecutive, 20 µm thick sections surrounding the injured area. Since our micro-CT analyses indicated that the injury spanned an area approximately 90 µm deep, our analysis represents almost half of the injured area. Our present results indicate that more comprehensive monitoring of the areas of interest and analyzing longer healing timelines are important to advance our knowledge of how the pulp tissue responds to injury. We also suggest that etching and the use of toxic pulp capping materials, such as calcium hydroxide, be avoided or minimized whenever possible in dental procedures, especially in rodent models, because these may damage the peptidergic afferents and impede repair processes.

In our model, the dentin proteome in the *Tgfbr2^cko^* and WT mice should be nearly identical due to the suppression of Cre recombinase until doxycycline was withdrawn during the experimental period. The dentin proteome has been shown to promote neurite outgrowth [67,68] and to stimulate odontoblast differentiation and/or tertiary dentin secretion [69,70]. The growth factors in dentin can be released into the pulp during endodontic treatments [71,72,73,74,75,76] and are being studied for their potential in regenerative endodontics. It is possible that the dentin similarities in our *Tgfbr2^cko^* and WT mice may be masking a differential response that would occur under other conditions. Since we did not apply acid or EDTA to release dentin proteins, future studies will be required to confirm this.

Previous reports suggest that inhibiting TGFβ receptor signaling disrupts the odontoblast layer and dentin secretion, leading to osteodentin production [31,38] rather than tubular dentin. Here, we show that tubular dentin secretion is possible from TGFβ-deficient cells. Interestingly, two reports showed that less porous dentin stains scarlet in Masson’s Trichrome [77,78], which we saw in the outer regions of dentin and tertiary dentin that were not evident in the H&E images. By focusing on the locations of red versus blue in tooth healing, it could be possible to better ascertain the dentin integrity and porosity and perform more subtle investigations of the dentin–pulp complex, such as in pulp regeneration in aged teeth or with implanted bone marrow stem cells. In order to develop better treatments for patients with skeletal defects due to disrupted/deficient TGFβ signaling, such as patients with Loeys–Dietz Syndrome, Marfan Syndrome [10,79,80], or diabetes [5,81,82], future investigations should focus on developing models where the fibroblasts and the dentin are both modified. To investigate how the dental pulp maintains the balance of wound healing without fibrosis in mature teeth, we recommend a timed deletion like what was performed in the present study. In addition, our system provides a model to investigate how TGFβ-inhibiting drugs may impact the dentition and how to overcome the side effects of these drugs.

A literature review indicated that a variety of timelines have been used in previous investigations of tertiary dentinogenesis after injury. The collection of samples ranged from 4 to 56 days post-injury [38,40,83,84,85], and tertiary dentin has been observed as early as 7 days when heat and acid etching were applied [40]. We did not see any tertiary dentin in our samples collected on days 4 or 8 after injury, which we initially attributed to our lack of heat with the low-speed drill and the lack of etching. However, we found our discrepancy curious, particularly since one report indicated that tertiary dentinogenesis is slower in the C57 strain [86], which we were utilizing. We therefore extended our analyses to 56 days. If we had completed our analyses at the planned 21-day time point, our conclusion would have been that *Tgfbr2* was necessary for reactionary dentinogenesis. Instead, we found a compensatory mechanism involving sensory afferent signaling that promoted equivalent levels of tertiary dentin secretion. Our results strongly support the need for longer-term studies in regenerative endodontics, particularly in the C57 mouse strain, to fully assess whether healing is prevented or simply delayed and to provide accurate identification of potential candidates for clinical translation. This is particularly relevant to dental clinicians given the research showing that TGFβ signaling plays a large role in regulating the dentin–pulp complex [25,28,31,35,87] and that disrupted TGFβ signaling can delay oral wound healing [88]. These studies also help us to understand the balance between TGFβ-driven wound healing and fibrosis, as well as suggesting that patients utilizing TGFβ-inhibiting drugs may need additional (longer-term) follow-up after endodontic treatments to ensure proper healing.

We previously demonstrated that several members of the semaphorin family (SEMAs) were downstream of *Tgfbr2* in the dental pulp and regulated neurite outgrowth in the developing molars [25]. It is possible that changes in the odontoblast expression levels of these chemoattractants in our *Tgfbr2^cko^* mice could be directly altering, i.e., prolonging, the neurite outgrowth during the injury response. When recombinant SEMAs were applied to mineralized tissues, they led to a range of results, from aggravating existing periapical lesions [89] to promoting reparative dentin formation in pulp capping experiments [90] and bone healing in fracture calluses and calvarial defects [91,92]. In addition, SEMA-regulated bone repair has been shown to be driven by the sensory nerves, rather than osteoblasts [93,94], indicating that there is complex neuronal–mesenchymal crosstalk during repair. Several reports also indicate that Semaphorin 7a (SEMA7a) plays a critical role in regulating TGFβ induced fibrosis [95,96,97,98], indicating that this signal (which is downstream of *Tgfbr2* in the pulp [25]) may be key to understanding how dental pulp homeostasis and healing occur. Mouse models of different semaphorin deletions, particularly SEMA7a, in mineralizing and innervating populations of the dental pulp should be evaluated to fully address the roles these chemoattractants play during reactionary and/or reparative dentinogenesis.

Interestingly, bone research has recently found that sensory afferents assist bone healing via CGRP signaling. For instance, bone healing around orthopedic implants can be attenuated with denervation of sensory nerves or knockdown of the genes encoding CGRP receptors, *Calcrl* or *Ramp1*. Conversely, upregulating the receptors with adenovirus-mediated overexpression (AdV-*Calcrl*) enhanced osteogenesis [99]. Another group observed increased CGRP around 3 days after a femoral fracture in mice, similar to the CGRP spike found in molars [42]. However, the CGRP receptors were not upregulated until 1-2 weeks post-injury. This indicated that the increased expression of CGRP and its receptors during bone healing were not aligned [100]. A follow-up study performed an ACL reconstruction where they injected hydrogel microparticles loaded with adenoviruses to silence or overexpress the CGRP receptors (adv-sh*Calcrl* or adv-*Calcrl*) in the bone tunnels. Their results showed striking reductions/increases in mineralization markers and bone volume following the silencing/overexpression of the CGRP receptors, similar to the findings of the orthodontic implant study [99]. This demonstrated that increasing the expression of CGRP receptors at the earlier time points, when CGRP levels peak, dramatically improves bone healing [101]. We hypothesize that the prolonged peak of CGRP in our *Tgfbr2^ck^*^o^ mice led to the CGRP being present when the receptor expression levels were higher, which allowed for additional mineralization signals in the absence of *Tgfbr2*. We suggest that more studies should be performed to address how CGRP signaling regulates the pulp tissue. Such studies would help to decipher how CGRP signaling might be manipulated as part of vital pulp therapies to enhance healing and vitality as it has been shown to do in skin [56], cornea [102], bone [99,103], and, most importantly, dental pulp [39,40,49,83].

## 4. Materials and Methods

### 4.1. Experiment and Specimen Preparation

#### 4.1.1. Mouse Model

All mouse experiments were approved by the OSU Institutional Animal Care and Use Committee. The *Tgfbr2^cko^* mice were described previously [25,26,27]. To inhibit Osx-Cre activity, *Tgfbr2^cko^* breeding cages were maintained on a doxycycline-enhanced diet (7012, 1 g/kg, TD.08826, Teklad, Madison, WI, USA). *Tgfbr2^cko^* mice were moved to standard chow two weeks before creating a shallow dentin injury (Figure 1A) to allow for *Tgfbr2* during the experimental period [104]. Wild-type (WT) C57BL/6J mice were maintained on standard chow.

#### 4.1.2. Dentin Injury and Mandible Harvest

Three-month-old mice were anesthetized with isoflurane, and with the aid of an endodontic microscope (Enova Illumination, Minneapolis, MN, USA), the cemento–enamel junction (CEJ) of the mesial mandibular first molar (M1; Figure 1B–D) was exposed. A shallow dentin injury was then created with a low-speed handpiece fitted with a #1/16 carbide round bur (Komet, Rock Hill, SC, USA, H1.314.003). At 4, 8, 21, and 56 days post-injury (dpi), the mice were injected with a ketamine/xylazine cocktail (1 g/10 g body weight; 25 mg/mL of ketamine, 2.5 mg/mL of xylazine, Covetrus North America, Portland, ME, USA), weighed, and transcardially perfused with cold PBS followed by 4% paraformaldehyde (PFA) to collect mandibles. For mice collected on 4–21 dpi, the right mandible was injured, and the left mandible served as a contralateral control. For those sacrificed at 56 dpi, the right and left mandibles were both injured for separate experiments. Dissected mandibles were post-fixed in 4% PFA for 2 h at room temperature.

### 4.2. Staining and Microscopic Observation Methods

#### 4.2.1. In Situ Hybridization (ISH) and Histological Staining

Paraffin-embedded tissues were sectioned coronally at 7 µm in preparation for ISH or histological staining. Active Sp7 transcription was identified by ISH, which was performed using the RNAscope RED kit (ACD, 322373) and an Sp7 probe (ACD, 403401) per the manufacturer’s instructions. The presence of Sp7 transcripts confirmed the *Tgfbr2* deletion in Osterix-Cre+ mice. Hematoxylin and eosin (H&E) and Masson’s Trichrome stains were applied according to standard protocols.

#### 4.2.2. Micro-CT Scanning and Analysis

Fixed hemi-mandibles were stored and scanned in 70% ethanol with a µCT 50 (Scanco Medical, Bassersdorf, Switzerland) at 70 kVp, 76 µA, with a 0.5 mm Al filter, 900 ms integration and 6 µm voxel dimension. DICOM files were calibrated to a standard curve calculated from five known densities of hydroxyapatite (mg/cm^3^ HA). The mandibles were oriented with the mid-sagittal plane of the tooth. The first mandibular molar’s dentin was segmented at 500–600 mg/cm^3^ HA and enamel was segmented at 1600 mg/cm^3^ HA. This segmentation map was applied for n = 5–6 sample size. The control mandibles were analyzed as described, and their respective segmentation maps were applied. Reconstructed images were analyzed using Analyze 14.0 (AnalyzeDirect, Overland Park, KS, USA), as described previously [105,106]. A region of interest (ROI) was created to encompass the largest region of tertiary dentin found in any of the samples (90 µm total), and this was applied to all injured samples. Control images are shown for reference. N = 6/genotype at 21 dpi, n = 5 for control and n = 6 for *Tgfbr2^cko^* at 56 dpi.

#### 4.2.3. Immunofluorescence, Confocal Imaging, and Image Analysis

Hemi-mandibles were decalcified in 10% EDTA and prepared for cryo-embedding as described previously [25]. Twenty-micron sections were permeabilized using 0.5% Tween-20 blocking solution, followed by application of the primary antibody (anti-calcitonin gene-related peptide [CGRP]; Immunostar, Hudson, WI, USA, 24112, 1:2000), secondary antibody (goat anti-rabbit IgG, Invitrogen, Waltham, MA, USA, 1:500), and DAPI. Negative control experiments without primary antibodies did not yield significant staining.

Confocal microscopy was used to image CGRP fluorescence in z-stacks (20 µm) in the area of injury and similar areas for uninjured (control) molars. The results were converted to maximum projection images for analysis using ImageJ 1.54h (National Institutes of Health, Bethesda, MD, USA). CGRP confocal images were then converted to 8-bit, autothresholded with the mean option, and the injury area (or similar area in control molars) was isolated for pixel quantification. When possible, two serial sections per animal (40 µm total) were used for quantification and statistical analyses.

### 4.3. Statistical Analysis Methods

A two-way mixed-effects analysis of variance (ANOVA) with Šídák’s multiple comparisons was used to assess weight changes after injury, and differences in dentin deposition in micro-CT were evaluated with a two-way mixed-effects ANOVA with Tukey’s multiple comparisons using GraphPad Prism 10.2.2 (GraphPad Software, accessed on 1 May 2024, www.graphpad.com). Significance was set at 5% for both ANOVAs.

Generalized estimating equations (GEEs) [58] were used to analyze CGRP sprouting over all time points for both injured and control mice, controlling for genotype (*Tgfbr2^cko^* or WT) and accounting for repeated measurements resulting from evaluating two sections from each sample. Since only injured M1s were investigated at 56 dpi, injured M1s were considered the reference group for modeling (See Supplemental Table S1). Significance from estimated effects was tested using a Wald test with a 5% significance level.

## 5. Conclusions

The aim of this study was to determine if the development neuro-pulpal crosstalk regulated by *Tgfbr2* in the dental pulp fibroblasts was recapitulated during dentin regeneration, i.e., reactionary dentinogenesis. To this end, we performed a timed deletion of *Tgfbr2* in the dental pulp mesenchyme immediately prior to a shallow dentin injury and quantified the levels of tertiary dentin and CGRP+ axon sprouting. Micro-CT analysis, H&E images, and Masson’s trichrome images all demonstrated that the tertiary dentin secretion was delayed in *Tgfbr2^cko^* mice but reached equivalence to the WT controls by two months. The levels of CGRP+ axon sprouting were similar in both groups at 4 days post-injury but were elevated for a longer duration in the *Tgfbr2^cko^* mice. This was in striking contradiction to the developmental studies showing that *Tgfbr2* deletion resulted in severely hypomineralized and hypoinnervated dentition. We suggest that when normal development is allowed, the peripheral nervous system can provide compensatory signals to assist healing, even in the presence of disease and/or TGFβ-inhibiting drugs. Given the current debates about TGFβ-driven fibrosis and therapeutics as well as the role(s) of CGRP in bone, tooth and overall health, we believe subsequent studies should address the CGRP ligand–receptor complex during repair of the dentin–pulp complex. These investigations are likely to provide information that will lead to better long-term outcomes for dental restorations and regenerative endodontics and may shed light on a variety of conditions related to TGFβ.

## Data Availability

The raw image data supporting the conclusions of this article will be made available by the authors on request.

## Supplementary Materials

The following supporting information can be downloaded at: https://www.mdpi.com/article/10.3390/ijms25136847/s1.
