# Supplementary material for: Impaired Tertiary Dentin Secretion after Shallow Injury in Tgfbr2-Deficient Dental Pulp Cells Is Rescued by Extended CGRP Signaling
Source: Int J Mol Sci. 2024 Jun 21;25(13):6847. doi: 10.3390/ijms25136847 (PMC11241056; doi:10.3390/ijms25136847)
Supplement: Supplementary file 1 [file ijms-25-06847-s001.zip › ijms-3039900-supplementary.pdf]

Supplementary Materials:

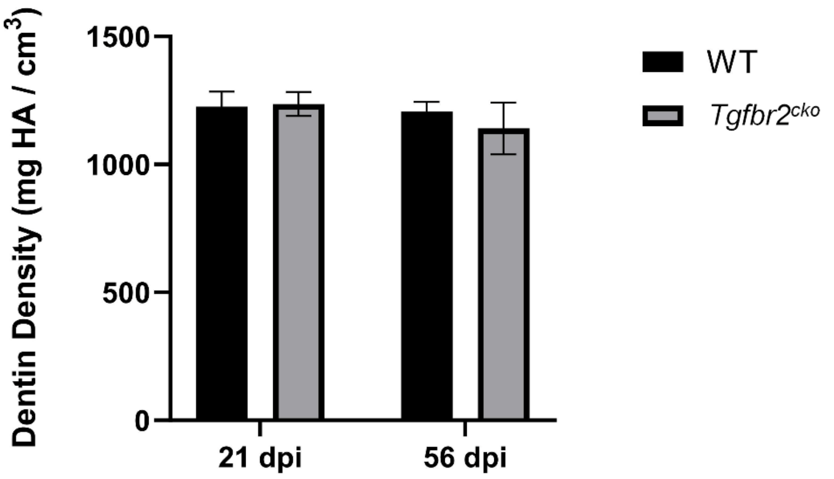

Figure S1. Micro-CT analysis of dentin density. No differences in dentin density were found at 21 or 56 dpi between WT and Tgfbr2^{cko} M1s.

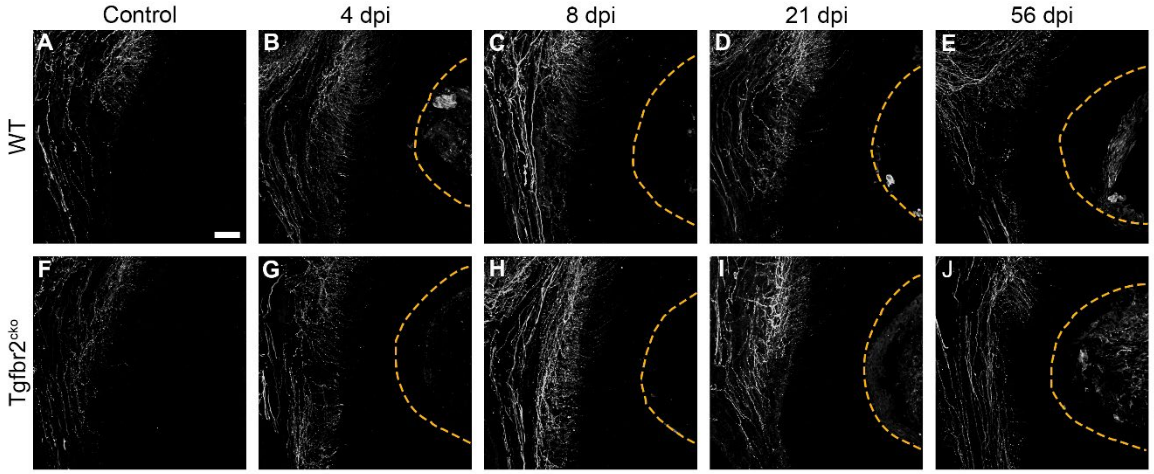

Figure S2. Isolated CGRP+ axon sprouting in response to injury. Timeline of isolated CGRP+ axon outgrowth (white) in uninjured, and 4, 8, 21, and 56 dpi WT (A-E) and *Tgfbr2^{cko}* (F-J) M1s, demonstrating divergent outgrowth patterns from 4-21 dpi between genotypes. Dotted yellow lines indicate areas of dentin injury. Scale bar in (A) = 50 μm.

Table S1. Results from the fitted GEE, accounting for repeated measures from evaluating two sections from each sample.

Term	Estimate	Std. Error	Statistic	*p* Value
Intercept	1,875,690	199,702	88.218	**<0.0001**
WT	355,742	272,975	1.698	0.1925
Ctl	−301,958	302,287	0.998	0.3178
8 dpi	92,072	308,701	0.089	0.7655
21 dpi	355,390	335,635	1.121	0.2897
56 dpi	−548,734	463,553	1.401	0.2365
WT:Ctl	−719,330	412,341	3.043	0.0811
WT:8 dpi	−560,670	413,896	1.835	0.1755
WT:21 dpi	−1,161,959	407,350	8.137	**0.0043**
Ctl:8 dpi	−93,531	414,278	0.051	0.8214
Ctl:21 dpi	−18,368	448,516	0.002	0.9673
WT:56 dpi	−824,827	521,689	2.5	0.1139
WT:Ctl:8 dpi	1,354,414	581,367	5.428	**0.0198**
WT:Ctl:21 dpi	921,794	576,407	2.557	0.1098

Reference group: *Tgfbr2^{cko}*, day 4, injured

Generalized Estimating Equations (GEE) is a method for fitting (generalized) linear regression models to clustered data, which yields from having more than one measurement per mouse. By using the Huber-White method to estimate standard errors, valid inferential results are expected even when the working correlation is misspecified. In particular, we considered an identity link, normal variance, and working independence. The injured group was used as reference (as opposed to the control) since data from 56 dpi was only available for injured mice. However, results are equivalent since only two groups (injured and control) were considered. Overall, A:B denotes the interaction term between factor A and factor B, and A:B:C denotes the 3-way interaction between factors A, B, and C. The statistically significant findings are described below:

- WT:21dpi: The change from 4 dpi CGRP to 21 dpi CGRP in wild type mice is significantly different than the change from 4 dpi CGRP to 21 dpi CGRP in *Tgfbr2^{cko}* mice. In other words, the effect of changing dpi from 4 to 21 is significantly different between genotypes, with a p-value of 0.00434.
- The difference in the change from day 4 dpi CGRP to day 8 dpi CGRP between wild type mice and Tgfbr2^{ck}o mice in control teeth is significantly different than the difference in the change from day 4 dpi CGRP to day 8 dpi CGRP between wild type mice and *Tgfbr2^{cko}* mice in injured teeth, with a p-value of 0.0198.

Taken together, this indicates that due to the long time period in which the axon sprouting is equivalent at the beginning and at the end, we found many similarities between the CGRP levels. However, we found that the timeline from 4 dpi to 21 dpi was different between the genotypes, indicating a differential healing response via CGRP.

Table S2. Testing for Interaction with Sex – analysis of Wald Statistic.

Term	Degrees of freedom	p Value
Type	1	0.0797
Treatment	1	0.1924
Dpis (4, 8 and 21)	2	0.1864
Sex	1	0.6392
56 dpi	1	**0.0008**
Type x Treatment	1	0.9848
Type x dpis	2	**0.0213**
Treatment x dpis	2	0.1164
Type x Sex	1	0.0599
Treatment x Sex	1	0.3428
dpi x Sex	2	**0.0116**
Type x 56 dpi	1	0.3653
Sex x 56 dpi	1	0.2191
Type x Treatment x dpis	2	0.0680
Type x Treatment x Sex	1	0.8755
Type x dpis x Sex	2	0.1694
Treatment x dpis x Sex	2	0.3038
Type x Sex x 56 dpi	1	0.7501
Type x Treatment x dpis x Sex	2	0.8910

Table S2 presents results from sequentially adding terms (first to last) to an extended factorial model that additionally accounts for sex, based on Wald tests. For a 5% significance level, we note that the only significant term involving sex is its interaction with dpi (for days 4, 8, and 21), and that all terms involving both sex and treatment (injured or control) are not significant, indicating that we do not have evidence that the effect of the injury in the CGRP is different between sexes.
As a thecinical note, the term 56 dpi appears separatly in Table S2 since it was accounted for as a separate variable (since data was only available for the injured group at this level and a full factorial model would not be identifiable).
